# A New Pixels Flipping Method for Huge Watermarking Capacity of the Invoice Font Image

**DOI:** 10.1155/2014/895063

**Published:** 2014-11-12

**Authors:** Li Li, Qingzheng Hou, Jianfeng Lu, Qishuai Xu, Junping Dai, Xiaoyang Mao, Chin-Chen Chang

**Affiliations:** ^1^Institute of Graphics and Image, Hangzhou Dianzi University, Hangzhou, Zhejiang 310018, China; ^2^Institute of Digital Media, Hangzhou Dianzi University, Hangzhou, Zhejiang 310018, China; ^3^University of Yamanashi, Yamanashi-ken 409-3866, Japan; ^4^Department of Information Engineering and Computer Science, Feng Chia University, Taichung 40724, Taiwan; ^5^Department of Computer Science and Information Engineering, Asia University, Taichung 41354, Taiwan

## Abstract

Invoice printing just has two-color printing, so invoice font image can be seen as binary image. To embed watermarks into invoice image, the pixels need to be flipped. The more huge the watermark is, the more the pixels need to be flipped. We proposed a new pixels flipping method in invoice image for huge watermarking capacity. The pixels flipping method includes one novel interpolation method for binary image, one flippable pixels evaluation mechanism, and one denoising method based on gravity center and chaos degree. The proposed interpolation method ensures that the invoice image keeps features well after scaling. The flippable pixels evaluation mechanism ensures that the pixels keep better connectivity and smoothness and the pattern has highest structural similarity after flipping. The proposed denoising method makes invoice font image smoother and fiter for human vision. Experiments show that the proposed flipping method not only keeps the invoice font structure well but also improves watermarking capacity.

## 1. Introduction

With the development of multimedia technology, more attention is drawn to the certification of digital media content in the aspects of security, confidentiality, authenticity, and integrity, which, as a result, promotes the development of information hiding and digital watermark technology.

Nowadays, a large number of documents such as financial, insurance, and patents have been transformed into digital documents and stored as binary image documents. To guarantee the certification appears on the invoice font image, binary image watermarking technique is applied. According to human visual system model, few changes to the pixels on a color image or a grey image would not be perceived by human eyes because of the large pixel value range and high redundancy [1–5]. However, binary images only have two colors (black and white) which means little redundancy. When changing the black (white) pixels of a binary image into white (black) ones, human eyes can catch the change easily. Therefore, it is more difficult to embed watermark into binary images than into color images and grey images.

At present, there are some watermarking algorithms on binary image [6–11]. These algorithms can be classified into two categories. One category is based on adjusting the structure information of image content. Tan et al. [12] proposed a binary image watermarking algorithm based on changing the direction of the stroke, but it is for Chinese character image. The other category is to flip the pixels of binary image, that is to embed watermark information by changing the black (white) pixels into white (black) ones. Wu and Liu [8] proposed a scoring standard to judge the “flappable” pixels and embedded watermark by flipping the pixels of the binary image. Zhao and Koch [13] proposed a method to embed watermark by changing the rate of numbers of the black pixels and white pixels in one block. Qi et al. [10] embedded watermark according to the multiplicative transformation model to modify black pixels numbers of character picture.

Most binary image watermarking algorithms based on flipping pixels would use the scoring standard proposed by Wu and Liu [8]. But Wu's method has some weakness because the flippable pixels are obtained by the connectivity and smoothness of pixels. Firstly, the structure information around pixels is not considered; secondly, when a huge number of pixels need to be flipped, Wu's method may not be able to meet the flipping quantity requirement; lastly, with the numbers of flipped pixels increasing, the flipped binary image would have some noises just like some burrs using Wu's method. This results in bad visual quality of the flipped binary image.

To solve those problems, we proposed a novel algorithm to flip pixels for the binary font image. It includes one novel scaling interpolation for binary image, one novel pixels flippable evaluation mechanism, and one novel denoising method based on gravity center and chaos degree. The scaling interpolation method ensures that the invoice font image keeps features well after scaling. The pixels flippable evaluation mechanism ensures that the pixels keep better connectivity and smoothness and keeps the pattern highest structural similarity after flipping. The proposed denoising method makes the flipped invoice font image smoother and fitter.

## 2. The Proposed Pixels Flipping Method for the Invoice Binary Image

**Figure 1:**
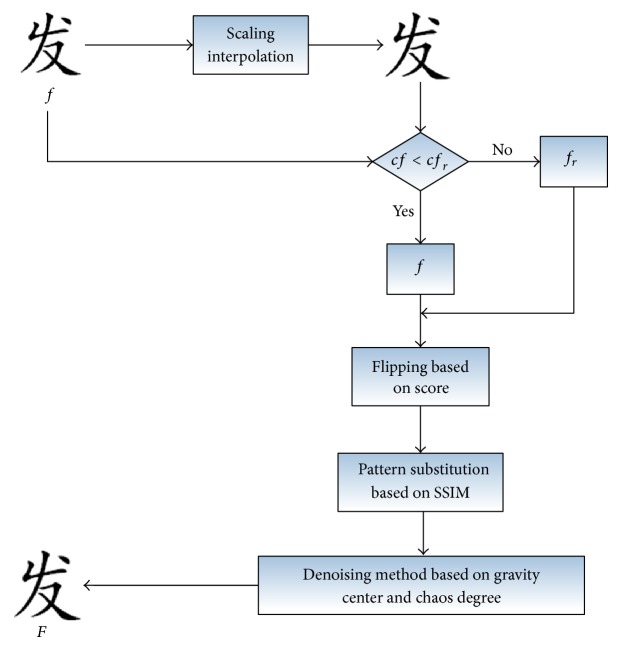
Algorithm flow chart: *f* represents the original image, *f*_*r*_ represents the binary font image after being processed using scaling interpolation method, and *cf*, *cf*_*r*_ represent the numbers of pixels that need to be flipped, respectively. *F* is the resulting binary font image.

The proposed pixels flipping method includes a few steps as shown in Figure 1. Step 1, by the small proportion of the scaling for the invoice font image to reduce the flipping number. The font binary image *f*_*r*_ is obtained by small proportion scaling interpolation of the original font binary image *f* based on the flipping number. Step 2 is to calculate the pixels flipping number *cf*, *cf*_*r*_ of *f* and *f*_*r*_, respectively, and choose the image as the final flipping object which has less flipping number. Step 3 is to choose and flip the flippable pixels based on proposed flippable evaluation mechanism. Step 4 is to denoise the watermarked invoice font binary image based on gravity center and chaos degree to get a better image quality.

The remainder of this section contains the following. In Section 2.1, the proposed scaling interpolation method for binary image is given. In Section 2.2, the proposed pixels flippable evaluation mechanism is given. In Section 2.3, the proposed pixels flipping strategy is given. In Section 2.4, the denoising method based on gravity center and chaos degree is proposed.

### 2.1. The Proposed Scaling Interpolation for Binary Image

The huge watermarking capacity of invoice binary image needs to flip large number of pixels. According to human visual system (HVS), few changes of pixels on an image cannot be perceived by human eyes. The invoice font image after scaling interpolation will add or reduce some black pixels, which effectively reduce the number of pixels to be flipped.

**Figure 2:**
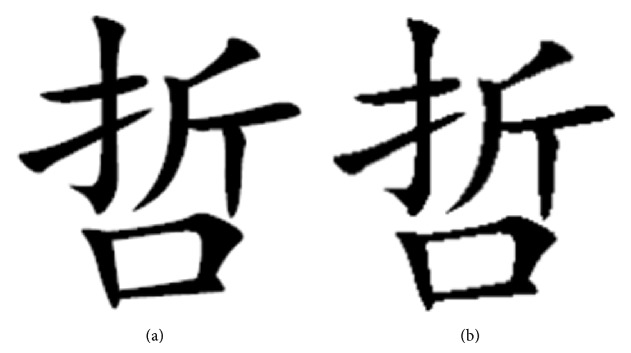
Comparison of the original image and the scaled one. (a) The original image; (b) the font image scaled by 102% using the bilinear interpolation algorithm.

The invoice font image after scaling keeps the original font structure features well, such as Figures 2(a) and 2(b). After scaling, Figure 2(b) keeps the whole font structure well except generating some noises which look like sawtooth though and the change is not sensitive. Therefore, before flipping the pixels, the font image needs scaling interpolation according to the ratio between numbers of pixels to be flipped and numbers of the font image's total black pixels.

Traditional interpolation methods include nearest-neighbor interpolation, bilinear interpolation, bicubic interpolation, and spline interpolation. The interpolation methods mentioned above can achieve well effect to image scaling when the image is colored or grey. A new pixel value between 0 and 255 can be obtained using interpolation methods mentioned above. But a binary image only has two color values 0 and 255, and any other values between 0 and 255 are not allowed. In addition, the sawtooth effect caused by traditional interpolation methods will lead to bad effect to the flappable pixels which will be address later. To resolve these two weaknesses, we proposed a new interpolation algorithm for binary images.

Here we used the way similar to nearest-neighbor interpolation and bilinear interpolation. The new pixel is obtained by interpolating the 4 adjacent pixels of the original pixel. According to the 4 adjacent pixels, the new pixel to be interpolated can be placed in five different locations, as follows.

**Figure 3:**
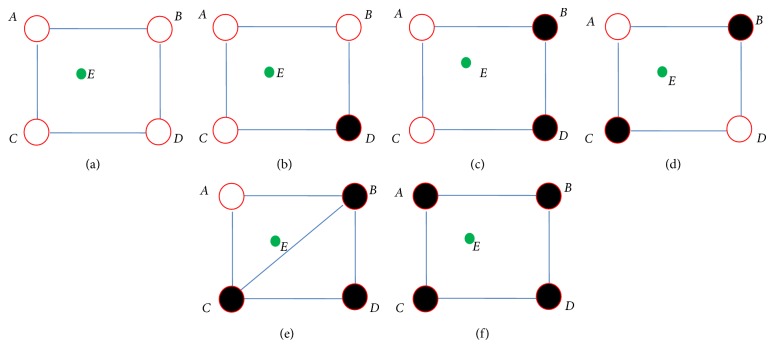
Classification of the five different locations: the green point *E* is the pixel to be interpolated. Letters *A*, *B*, *C*, and *D* represented by red circles are four pixels around *E*. (a) Four white pixels around; (b) three white pixels around; (c) and (d) two white pixels and two black ones around; (e) one white pixel and three black ones around; (f) four black pixels around.

1. Four pixels around are all white. See Figure 3(a).
2. Three pixels are white among the four pixels around. See Figure 3(b).
3. Two pixels are white among the four pixels around. See Figures 3(c) and 3(d).
4. Only one pixel is white among the four pixels around. See Figure 3(e).
5. Four pixels around are all black. See Figure 3(f).

We applied different strategies to acquire the pixel value to be interpolated aiming for the five cases above.

In case (1), pixel *E* has four white pixels around it, so we think *E* in this case should be placed in white flat area and its value must be white. Otherwise, the binary image may show some noises.

In case (2), pixel *E* is not in a flat area but the number of white pixels is larger than that of black ones, so pixel *E* must be white. If *E* is black, *E* will become a noise with the diagonal *BC* being white.

In case (3), when the four pixels are distributed like in Figure 3(c), we think that the font image's stroke has the direction of *BD*, and *BD* is the edge of the font from black to white. If we set *E* to black pixel, the stroke direction of the original font image will be destroyed, so pixel *E* must be white. When the four pixels are distributed like in Figure 3(d), we are not able to judge the direction of the stroke, so we find a pixel which is nearest to pixel *E* from pixels *A*, *B*, *C*, and *D*, and then set pixel *E*'s value to its nearest one's value.

In case (4), there is only one white pixel around pixel *E*, as shown in Figure 3(e). Pixel *E* is not in a flat area but in the edge of the stroke from black to white. In this case, we think that the stroke of font image has the direction of diagonal *BC*, and whether *E*'s value is black or white must be determined by the position of *E*—whether it is inside or outside of triangle Δ*BC* *D*, so we just need to judge that *E* is in bottom right or top left of line *BC*. This can be determined by computing the distance of *E* to *A* and *E* to *D*. The Euclidean distance of *E* to *A* and *E* to *D* is defined as dist⁡(*E*, *A*) and dist⁡(*E*, *D*) separately. *F*(*E*) is defined as the pixel value to be interpolated. Their relations are as follows:

(1) $$\begin{array}{c} F\left(E\right)=\left\{\begin{array}{cc} 255, & \mathrm{dist}\left(E,A\right)<\mathrm{dist}\left(E,D\right)\\ 0, & \mathrm{dist}\left(E,A\right)\geq \mathrm{dist}\left(E,D\right). \end{array}\right. \end{array}$$

Case (5) is similar to case (1), and pixel *E* is set to black.

**Figure 4:**
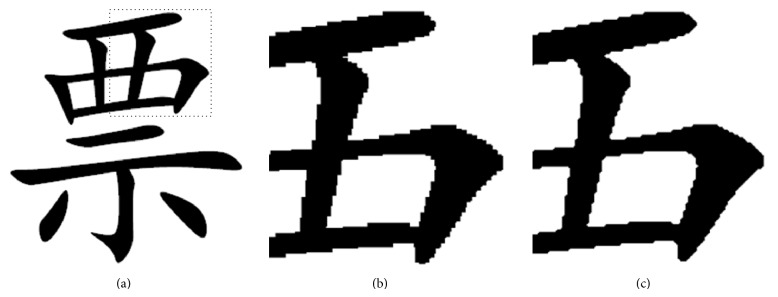
Comparison of traditional interpolation method and our method. Because the image resolution is too high after magnifying, we only showed the dotted line area of original image: (a) original image; (b) nearest-neighbor interpolation method; (c) our proposed scaling interpolation method.

The interpolation effect acquired from the method above can reduce sawtooth effect efficiently, and it is specially fit for scaling interpolation of font image. Only nearest-neighbor interpolation among traditional interpolation methods can be applied to binary image. So we compare our method against the nearest-neighbor interpolation method and simultaneously magnify the image by two sizes. We only showed a partial area of the font image because the image resolution is too high after magnifying. The comparison result shows that our method can reduce sawtooth effect efficiently, as shown in Figure 4.

### 2.2. The Proposed Pixels Flippable Evaluation Mechanism

Wu's method chooses the flippable pixels only according to the connectivity and smoothness of pixels without the structure information. When a large number of pixels need to be flipped, it may not be able to choose enough flipping quantity. To solve those problems, we proposed a novel flippable evaluation mechanism to determine the pixels to be flipped and then only select those flippable pixels to be flipped. The flippable evaluation mechanism includes the score of connectivity and smoothness and the 3∗3 pattern substitution based on SSIM, and the pixels which have higher score are flippable but to those pixels which have lower score we search the 3∗3 pattern which has the maximum SSIM to substitute the original pattern and the pattern is flippable.

#### 2.2.1. Wu's Method on the Score of Pixels

**Figure 5:**
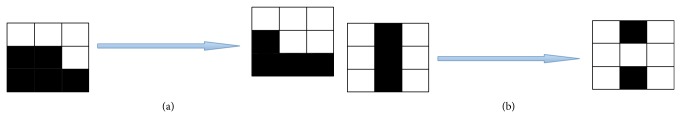
Two examples of flipping center pixel in 3∗3 window pattern, and (a) has better visual quality than (b) when flipping by using Wu's method.

Binary images only have two values, white and black which are denoted by 0 and 1, respectively, so the visual quality can be reduced greatly even if one value is changed. The binary image watermarking method is generally based on the modified pixels of the boundaries. Wu's method gives the score of the flippable pixels based on the smoothness and connectivity of the 3∗3 pattern window. The method determines the scores dynamically by observing the smoothness and connectivity. The smoothness is measured by the horizontal, vertical, and diagonal transitions in a local window (3∗3), and the connectivity is measured by the number of the black and white clusters. For example, Figure 5(a) has better visual quality when flipping the center pixel than Figure 5(b) using Wu's method.

**Figure 6:**
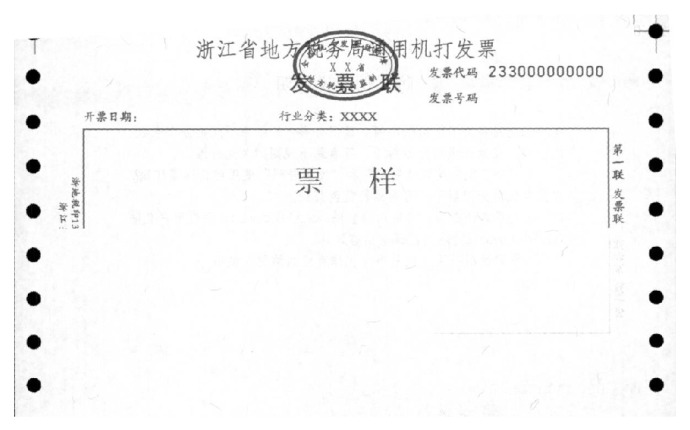
The invoice image.

**Figure 7:**
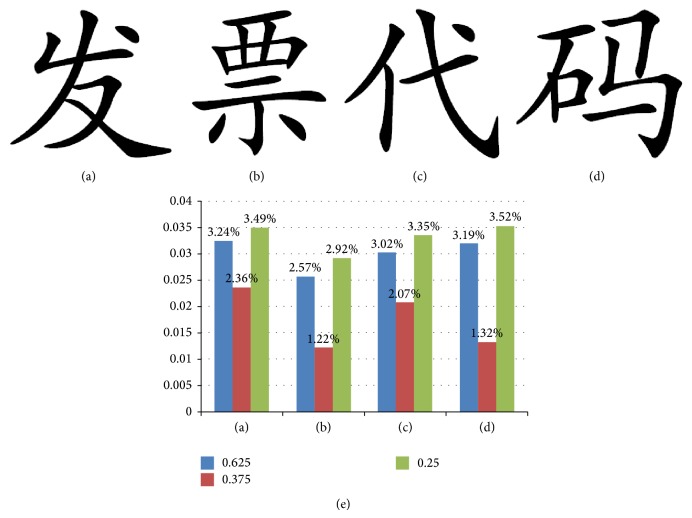
The summary of score ratio in four most-commonly used font binary images in invoice. In (e), the horizontal axis denotes the different font binary image and the vertical axis is the ratio in total pixels of this font binary image.

The higher score pattern indicates higher priority to flip the center pixel because of keeping good smoothness and connectivity after flipping. Using Wu's method, we flip those flippable pixels whose scores are greater than or equal to 0.3 to ensure the flipped image quality. We summarized the ratio of different score pixels in four most-commonly used invoice font binary images as shown in Figure 7. The invoice is shown in Figure 6.

It is concluded that the sum of ratio of 0.625, 0.375, and 0.25 in the four font binary images are 9.09%, 6.71%, 8.44%, and 8.03%, respectively. The ratio of flappable pixels in binary image is less than 10%. Therefore, when there are more than 10% pixels to be flipped, we cannot find enough flappable pixels using Wu's method. Moreover, Wu's method does not consider the structural of window pattern when flipping. So, in this paper, we adapted the 3∗3 window pattern substitution based on SSIM.

#### 2.2.2. The Proposed Pattern Substitution Based on SSIM

Wang et al. [14] introduced an alternative complementary framework for quality assessment based on the degradation of structural information. Based on that, a measure of structural similarity (SSIM) that compares local patterns of pixel intensities that have been normalized for luminance and contrast.

The SSIM can be described as

(2) $$\begin{array}{c} S\left(x,y\right)=f\left(l\left(x,y\right),c\left(x,y\right),s\left(x,y\right)\right). \end{array}$$

*x* and *y* are two images. The *S*(*x*, *y*) is the structural similarity of *x* and *y*.  *l*(*x*, *y*) is the luminance comparison function between *x* and *y*, *c*(*x*, *y*) is the contrast comparison function, *s*(*x*, *y*) is the structure comparison function, and *f*(·) is the combination function. The three comparison functions are defined as follows:

(3) $$\begin{array}{c} l\left(x,y\right)=\frac{2u_{x}u_{y}+C_{1}}{u_{x}^{2}+u_{y}^{2}+C_{1}},\;C_{1}={\left(K_{1}L\right)}^{2}, \end{array}$$

(4) $$\begin{array}{c} c\left(x,y\right)=\frac{2\sigma_{x}\sigma_{y}+C_{2}}{\sigma_{x}^{2}+\sigma_{y}^{2}+C_{2}},\;C_{2}={\left(K_{2}L\right)}^{2}, \end{array}$$

(5) $$\begin{array}{c} s\left(x,y\right)=\frac{2\sigma_{xy}+C_{3}}{\sigma_{x}\sigma_{y}+C_{3}}. \end{array}$$

*u*_*x*_ and *u*_*y*_ are the mean intensity of *x* and *y*, respectively, *L* is the dynamic range of the pixel value, and *K*_1_ and *K*_2_ are the two parameters which are far less than 1. *σ*_*x*_ and *σ*_*y*_ are the standard deviation of *x* and *y*, correlation coefficient between *x* and *y*.

Finally, combine the three comparisons of (3), (4), and (5) and obtain the similarity measure SSIM index between signals *x* and *y*:

(6) SSIMx,y=lx,yα·cx,yβ·sx,yγ,α>0, β>0, γ>0.

The value of SSIM is between 0 and 1; the larger the value is, the higher the similarity of the two signals is.

**Figure 8:**
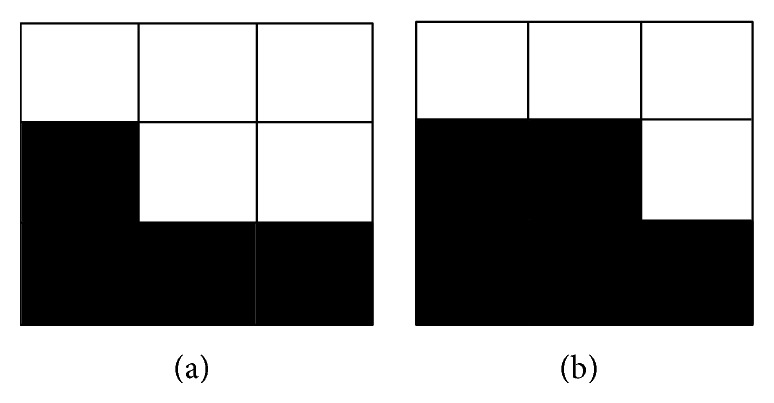
The two 3∗3 patterns: (a) the pattern *P*1 which has four black pixels; (b) the pattern *P*2 which has five black pixels. The pattern was calculated by SSIM, so *P*1 and *P*2 have the highest similarity than other patterns.

The SSIM not only is the method to evaluate two images but also gives us the idea of how to flip pixels. In this paper, we define the 3∗3 local window in binary image pattern. As shown in Figure 8, the pattern *P*1 has 4 black pixels; if we add black pixels to *P*1, we can calculate the SSIM of all the 3∗3 patterns where its black pixels count greater than 4 and choose pattern *P*2 which has the maximum SSIM value to substitute pattern *P*1.

### 2.3. The Proposed Pixels Flipping Method for Huge Watermarking Capacity

Assuming the original font binary image is *f*, the resulting image *F* is obtained by flipping Δ pixels of *f*. The quantity Δ of flipping pixels is determined by watermarks. The novel pixels flipping method for huge watermarking capacity is as follows.

*Step 1*. Scale the image *f* using our proposed interpolation method and determine the original font binary image or the scaled font binary image to be flipped.

The black pixels count of *f* is *n*; the black pixels count of *F* is *N* = *n* + Δ. The scaling ratio *α* based on *n* and Δ is estimated using the following formula:

(7) $$\begin{array}{c} \alpha =\frac{n+\Delta }{n}. \end{array}$$

*f* is scaled by using our proposed interpolation method according to the ratio *α*, the scaled font binary image is denoted by *f*′, and the black pixels count of *f*′ is *n*′. The distance between *n* and *N* is calculated. The distance between *n*′ and *N* is calculated, respectively:

(8) $$\begin{array}{c} \mathrm{dist}\left(n,N\right)=\left|N-n\right|,\\ \mathrm{dist}\left(n^{\prime },N\right)=\left|N-n^{\prime }\right|. \end{array}$$

*Step 2*. Select the smaller to be flipped by comparing dist⁡(*n*, *N*) and dist⁡(*n*′, *N*):

(9) $$\begin{array}{c} f=\left\{\begin{array}{cc} f, & \mathrm{dist}\left(n,N\right)\leq \mathrm{dist}\left(n^{\prime },N\right),\\ f^{\prime }, & \mathrm{dist}\left(n,N\right)>\mathrm{dist}\left(n^{\prime },N\right). \end{array}\right. \end{array}$$

*f* is the one to be flipped. The resulting flipping quantity *C* is obtained by the following formula:

(10) $$\begin{array}{c} C=\left\{\begin{array}{cc} N-n, & \mathrm{dist}\left(n,N\right)\leq \mathrm{dist}\left(n^{\prime },N\right),\\ N-n^{\prime }, & \mathrm{dist}\left(n,N\right)>\mathrm{dist}\left(n^{\prime },N\right). \end{array}\right. \end{array}$$

*Step 3*. Flip pixels based on the pixels scoring of smoothness and connectivity on *f*. Based on the threshold *T* = 0.3, sort all the pixels point by the scoring and flip the pixels which score greater than *T*. If *c*_1_ pixels point's score is greater than *T*, then there are *C* − *c*_1_ pixels to be flipped.

When there are a lot of pixels that need to be flipped, those sorted pixels whose scores are less than or equal to *T* are of sequential pattern substitution until achieving the flipping quantity.

### 2.4. The Proposed Denoising Method Based on Gravity Center and Chaos Degree

The flipped invoice font image which is generated by pixels flipping and pattern replaced by SSIM may produce burr or hole noises. In order to further reduce noises, we proposed an effective denoising method of pixels moving based on the gravity center and chaos degree of pattern.

The gravity center of an image is the center of the weight of the image. The weight of the binary image is the count of its black pixels; therefore, the black pixels distribute around the center of the gravity. The black pixels gather around the center of gravity, so the nearer the center of gravity of area is not allowed to have the appearance of white pixels. But before that, there should be one mechanism to judge whether the pixel in binary font image is noisy or not. So, we brought up the concept of chaos degree in pattern.

Assuming that the black pixels value in a binary image is 0 and white pixels value is 1, the chaos degree is the sum of difference between the pixels and its adjacent pixels of all the pixels in pattern where its size is 3∗3. The greater the chaos degree is, the more disorderly the pattern is. In this section, the patterns are noise patterns if their chaos degree is equal to or greater than 5. The definition of the chaos degree is as follows:

(11) SP=∑i=13 ∑j=23Pi,j−Pi,j−1 +∑j=13 ∑i=23Pi,j−Pi−1,j.

**Figure 9:**
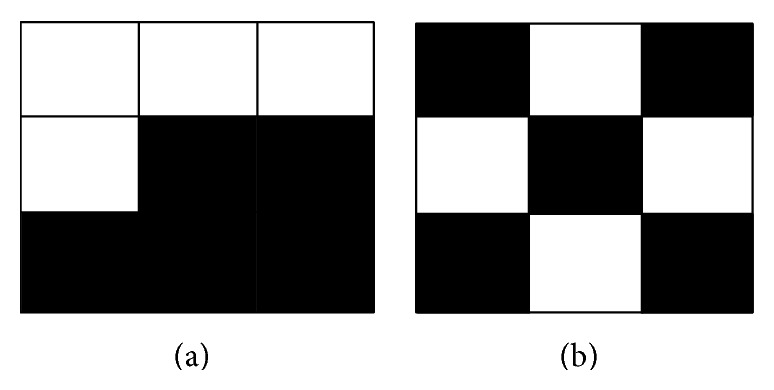
Two examples of calculating chaos degree: (a) its chaos degree is 4 and this pattern has a good quality; (b) its chaos degree is 12 and this pattern is a noise pattern and will bring about noise.

*P* is the pattern where its size is 3∗3 in binary image and *S*(*P*) is the chaos degree. In Figure 9, the two examples of calculation of chaos degree are given and the pattern in Figure 9(a) has good quality and will not bring about noise because its chaos degree is 4, but the chaos degree of the pattern in Figure 9(b) is 12; it is very messy in visual and this noise pattern must bring about burr or hole noise in font binary image.

Based on the proposed chaos degree, we can denoise the font binary image as follows.

*Step 1*. Calculate the chaos degree and find out the noise 3∗3 pattern *p*, the coordinate center of *p* as the center outward expansion to form a wider range pattern *p*′; for example, the size of *p*′ can be 5∗5, 7∗7, or 9∗9;

*Step 2*. Calculate the gravity center of *p*′, assuming the coordinate of gravity center is (*x*, *y*); the equation is as follow:

(12) $$\begin{array}{c} x=\frac{\sum_{i=1}^{h}\sum_{j=1}^{w}i*\left(1-p^{\prime }\left(i,j\right)\right)}{\sum_{i=1}^{h}\sum_{j=1}^{w}\left(1-p^{\prime }\left(i,j\right)\right)},\\ y=\frac{\sum_{i=1}^{h}\sum_{j=1}^{w}j*\left(1-p^{\prime }\left(i,j\right)\right)}{\sum_{i=1}^{h}\sum_{j=1}^{w}\left(1-p^{\prime }\left(i,j\right)\right)}. \end{array}$$

*Step 3*. Calculate the distance of gravity center (*x*, *y*) for each pixels point in pattern *p*′:

(13) $$\begin{array}{c} d_{\left(i,j\right)}=\sqrt{{\left(i-x\right)}^{2}+{\left(j-y\right)}^{2}}. \end{array}$$

Exchange the positions of the two points which have maximum distance black pixel and minimum distance white pixels to gravity center, respectively. By moving the black pixel which has the maximum distance to gravity center of *p*′ to the position of white pixels which have the minimum distance, it ensures that the nearer the center of gravity of area is not allowed to have the appearance of white pixels. The process is repeated until the chaos degree of pattern *p* falls below 5 or it cannot fall below 5 after having been repeated three times.

**Figure 10:**
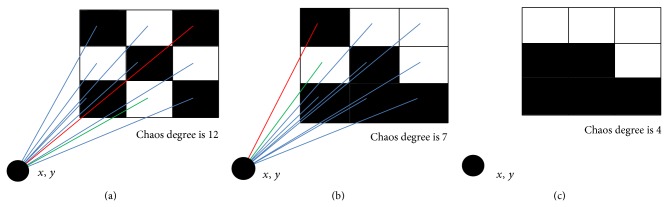
Denoise examples based ongravity center and chaos degree. The black point *x*, *y* is the gravity center of pattern *p*′. The line denotes the distance of each pixel to *x*, *y*, in which the red line is the maximum distance of black pixel to gravity center and the blue line is the minimum distance of white pixel to gravity center: (a) the chaos degree is 12; by moving the black pixel which has the maximum distance to white pixel which has the minimum distance, we get pattern (b) whose chaos degree is 7; by moving the black pixel, we get the pattern (c) whose chaos degree is 4, less than 5.

This denoise method can make the strokes of font binary image smoother and also fitter. The burr and hole noise in binary image can be effective reduced.  Figure 10 gives an example of denoise in one pattern; we can see that the pattern with chaos degree 12 can be reduced to 4 in three steps and the resulting pattern has a good quality and is fitter for the stroke of font binary image.

**Figure 11:**
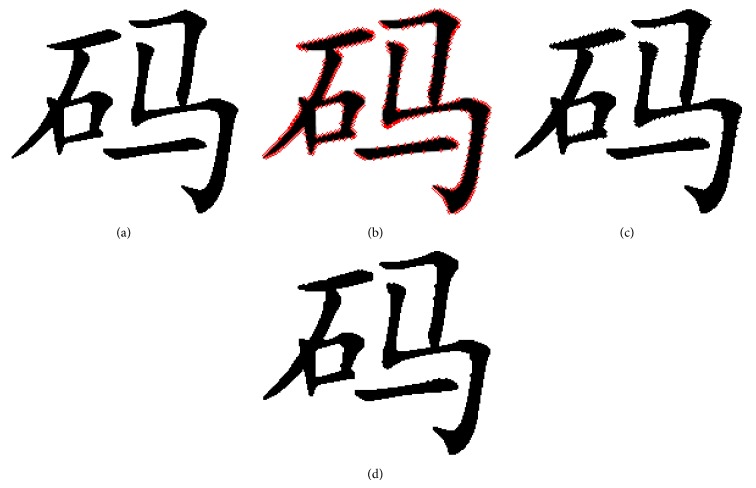
The application example of the proposed denoising method: (a) the original font binary image, black pixels count is 6335; (b) the position of the pixels flipping based on pixel score; the red points denote flipping position, flipping 500 pixles; (c) the image after pixels flipping; (d) the font binary image after denoising based on gravity center and chaos degree.

In Figure 11, it is concluded that the proposed denoising method based on gravity center and chaos degree can effectively reduce the burr and hole noise and make the font binary image smoother and fitter.

## 3. Experimental Results and Comparisons

A lot of experiments are conducted and the results show that the proposed algorithm is effective. In this section, we give the experiments results and the comparisons. Our comparisons focus on the image quality and pixels flipping capacity with Wu's method.

Wu's flipping method is an effective flipping method in binary image, but there are still some weaknesses.

1. It only considers the smoothness and connectivity but does not take into account the structure information around the flipping pixels.
2. When there are many pixels to be flipped, some burr or hole noises may occur.
3. When there are many pixels that need to be flipped, it may not be able to meet the flipping quantity requirement.

**Figure 12:**
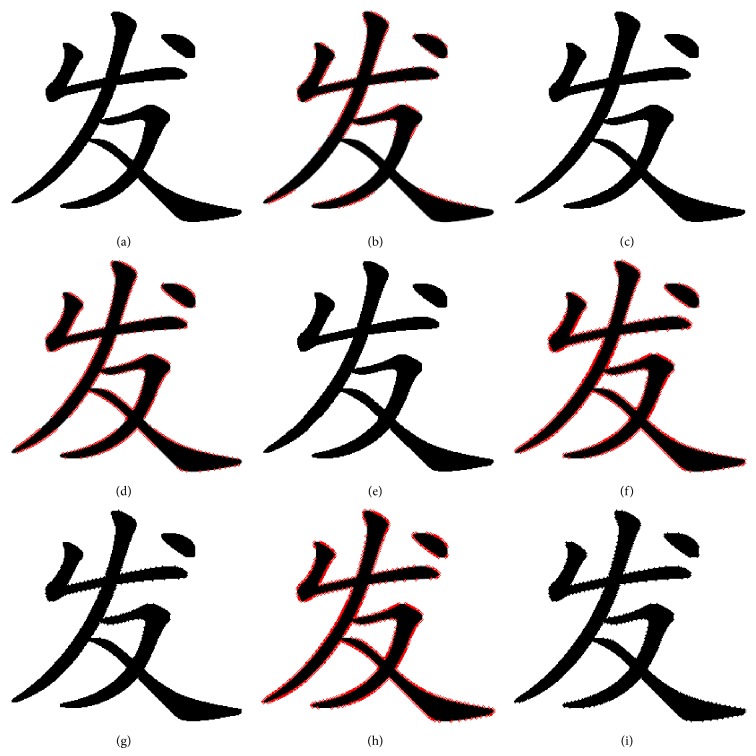
Flipping based on score of smoothness and connectivity. The black pixels count of the original font binary image is 12270; we flipped the original font binary image in every 300 pixels: (a) the original font binary image; (b) flipping position of adding 300 pixels in original font binary image; the red point denotes the flipping position; (c) the font binary image after flipping 300 pixels in original image; (d) flipping position of adding 600 pixels; (e) the font binary image after flipping 600 pixels in original image; (f) flipping position of adding 900 pixels; (g) the font binary image after flipping 600 pixels in original image; (h) flipping position of adding 900 pixels, but the number of flappable pixels is less than 1200; (i) the font binary image after flipping 1200 pixels in original image, but it just flipped 1116 pixels in real.

According to Wu's method, we flipped the font binary image in Figure 12(a) with a different pixel flipping quantity.

**Figure 13:**
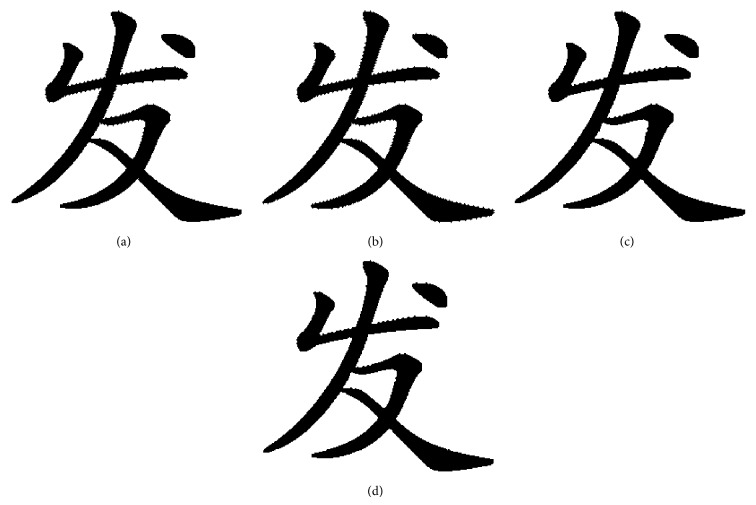
Denoising using the proposed based on gravity center and chaos degree: (a) the font binary image after flipping 900 pixels; (b) the font binary image after flipping 1200 pixels; (c) the font binary image after denoising which flipped 900 pixels; (d) the font binary image after denoising which flipped 1200 pixels.

It proved that the boundaries of the font stroke have a lot of burr when flipping count is more than 900 which results in greatly reduced visual quality. The proposed denoising method based on gravity center and chaos degree can reduce these noises. Denoise the font binary images after score as shown in Figure 13.

**Figure 14:**
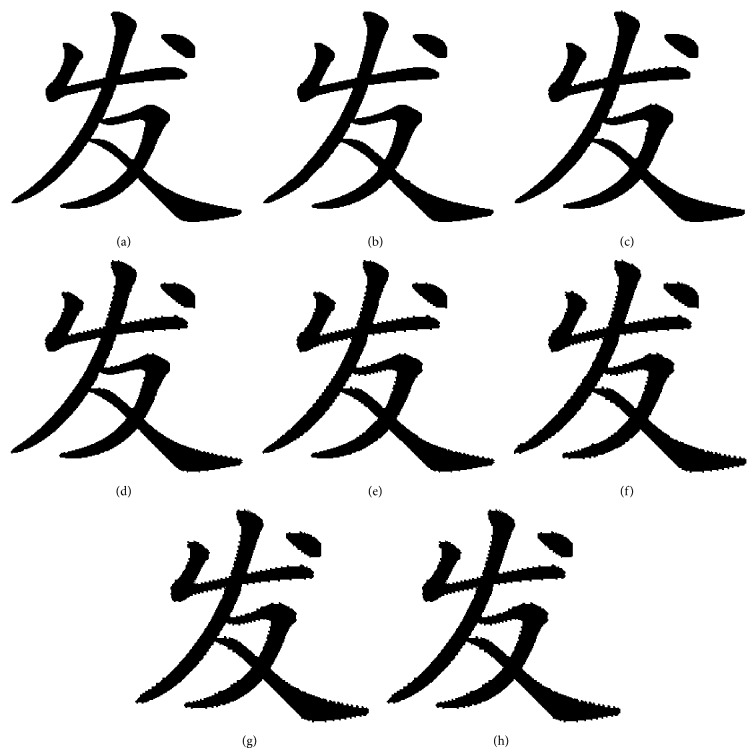
The flipped font binary image using our proposed flipping strategy: (a) flipping 300 pixels; (b) flipping 600 pixels; (c) flipping 900 pixels; (d) flipping 1200 pixels; (e) flipping 1500 pixels; (f) flipping 1800 pixels; (g) flipping 2100 pixels; (h) flipping 2400 pixels.

When flipping quantity is more than 1200, the number of flappable pixels is less than 1200. So it cannot work when flipping more than 1200 pixels unless it repeats flipping. The flipping method proposed can increase the capacity of flipping count and the pattern substitution based on SSIM ensures the structural similarity of font binary image. The proposed pixels flipping method is tested in Figure 14.

In Figure 14, the quality of flipping is twice as large as good as Wu's. The font binary images have better visualization compared to Wu's at same flipping quality, such as Figure 12(g) compared to 14(c) and 12(i) compared to 14(d).

**Figure 15:**
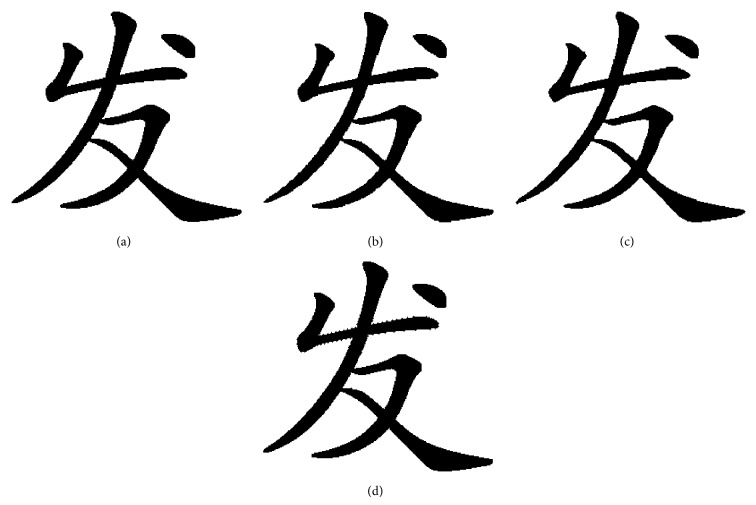
Flip the scaled font binary image; flipping count is 800 pixels: (a) original font binary image; (b) scaled font binary image which used our proposed interpolation algorithm; (c) flipped font binary image using our flipping strategy; (d) flipped font binary image using Wu's method.

The proposed flipping method can dynamically determine the object which is pixels flipping is original font binary image or the scaled font binary image which is using our interpolation algorithm. For example, when flipping 800 pixels the scaled font binary image was chosen as the flipping object. This method can increase the capacity of flipping efficiently as shown in Figure 15.

We can clearly see that Figure 15(c) has better visual quality than Figure 15(d) and is fitter for human vision.

## 4. Conclusion

We proposed a new pixels flipping method for huge watermarking capacity of invoice font image, including the proposed scaling interpolation of the binary image, pixels flippable evaluation mechanism, and the proposed denoising method based on gravity center and chaos degree. The proposed scaling interpolation method ensures that the font image keeps features well after scaling. The pixels flippable evaluation mechanism ensures that the pixels after flipping keep better connectivity and smoothness and the pattern after substitution has the highest structural similarity. The proposed denoising method makes the font binary image smoother and fitter for human vision. The experiment shows that our proposed flipping method not only keeps the font structure feature but also has more watermark embedding quantity.
